# Our New York Letter

**Published:** 1888-12

**Authors:** 


### OUR NEW YORK LETTER.

NEURASTHENIA—DISTINGUISHED MEDICAL VISITORS—COUNTER-PRESCRIBING—JAY GOULD'S PHYSICIAN.

*To the Editors Atlanta Medical and Surgical Journal:*

At a recent meeting of the Academy of Medicine, Dr. Landon Carter Gray read a paper before the Section in Practice of Medicine, on Neurasthenia — its Differentiation and Treatment. The general practitioner, he said, paid little attention to the subject of functional nervous diseases, as their study was of rather recent date. A correct classification of these troubles was much needed, and this, he said, explained the success of Dr. George M. Beard's book in Germany; but he could not agree with this gentleman regarding the frequent occurrence of true neurasthenia. He divided neurasthenia into three groups, viz., the reflex, the lithæmic and the simple. The first variety was caused by reflexes from non-nervous viscera, and also certain vicious habits. Lithæmia was the cause of the second variety. The third form was the one in which true nervous prostration existed. After referring at some length to the characteristic symptoms of these different varieties, he said that neurasthenia was also liable to be confounded with the early stages of a number of nervous diseases, among which were mentioned the different kinds of insanities, hysteria, anginoid attacks and locomotor ataxia. Referring to prognosis, he said it depended largely on the nature of the reflex cause, and the ability of being able to properly treat it. To treat the reflex variety, a removal of the cause would be necessary to bring about a cure.

In the lithæmic variety he recommended laxatives, nitro-muriatic acid and rest. Rest he considered very important in all cases, and there was, he said, very little danger of getting too much of it. The food should be looked after, and starches and nitrogenous substances prohibited for a period, after which the starches should be permanently reduced one-half. During the warm weather he had found it beneficial to prohibit nitrogenous food for several weeks. In treating true neurasthenia, he recommended the plan of Weir Mitchell, which included rest, massage, electricity and forced feeding. In addition to this he suggested iron and malt extract.

In the discussion which followed, Dr. W. R. Birdsall expressed some doubt as to whether the word neurasthenia should be used at all, as it seemed like a cloak for the physician's ignorance. He admitted that neurasthenia was an element in certain diseases, and would favor using the term in this limited sense. In functional disorders nervous symptoms were often prominent, owing to an instability of the nervous system which the patient inherited. This defect in the nervous system might also be acquired, and errors of refraction could give rise to neurasthenic symptoms. Referring to Dr. Gray's division of the subject, he believed that most cases would come under the lithæmic variety. Sexual excess he believed to be a frequent cause of neurasthenia. The employment of rest or exercise should be decided according to the nature of each individual case.

Dr. W. H. Thompson said he abominated the word neurasthenia, although it sounded very well before patients. He would divide cases into those of nervous debility, virtual starvation, overstrain and self-poisoning. Even when three meals daily were eaten, starvation with neurasthenic symptoms might occur. Worriment caused over strain. The self poisoning came from the intestines, by decomposition occurring and absorption of the poisonous elements into the circulation. These latter causes, he said, were called by Dr. Gray the lithæmic. In diagnosis, great reliance could be placed on the pulse and character of the urine.

Cases resulting from starvation and over-strain showed a weak, compressible pulse, with neutral or alkaline urine of low specific gravity. In the self-poisoning variety, there existed a high tension pulse, and the urine acid and of high specific gravity.

These lithæmic patients, he said, were always cured by camping out—a remedy which he had never known to fail.

Dr. A. Jacoby said that the word neurasthenia applied to a group of symptoms, the cause of which should be ascertained. Contrary to Dr. Gray's statement, he said that this group of symptoms had been described by several writers on the continent before Dr. Beard wrote.

In closing the discussion, Dr. Gray said that in some of the cases referred to under this name a pathological lesion might be found, but he believed there were instances where no lesion existed, the trouble being functional, and here the term neurasthenia was required. He had seen cases where this diagnosis was made, and yet after death no lesion could be found.

Receptions to our foreign medical visitors still continue to be the order of the day. Dr. David Ferrier and Mr. Victor Horseley have had a dinner given in their honor at the University Club by a number of prominent physicians. Dr. Sansom was also tendered a reception by Dr. Andrew H. Smith, at which a large gathering of well-known professional men was present. The last affair of this kind was given in honor of Dr. W. O. Priestley, of London, by Dr. T. Gaillard Thomas.

Although druggists here still continue to prescribe over the counter, they sometimes come to grief. A woman recently entered a drug-store complaining of feeling unwell, whereupon the proprietor looked at her tongue and prescribed for her supposed trouble. An agent of the County Medical Society then sent his wife to the same store to get doctored, and she had no difficulty in obtaining advice and also a bottle of medicine. At this stage the druggist was arrested, and in the Court of Special Sessions fined $50 for his illegal prescribing.

It is stated that the physician of Mr. Jay Gould receives $20.000 a year for his services, which have been contracted for for twenty years, or up to the time of Mr. Gould's demise.

A. F.

*New York, November 8th, 1888.*

